# Extracellular vesicles enriched with amylin receptor are cytoprotective against the Aß toxicity *in vitro*

**DOI:** 10.1371/journal.pone.0267164

**Published:** 2022-04-14

**Authors:** Rania Soudy, Ryoichi Kimura, Wen Fu, Aarti Patel, Jack Jhamandas

**Affiliations:** 1 Department of Medicine (Neurology), Neuroscience and Mental Health Institute, University of Alberta, Edmonton, Alberta, Canada; 2 Faculty of Pharmacy, Cairo University, Cairo, Egypt; 3 Center for Liberal Arts and Sciences, Sanyo-Onoda City University, Yamaguchi, Japan; Nathan S Kline Institute, UNITED STATES

## Abstract

Extracellular vesicles (EVs) are double membrane structures released by all cell types with identified roles in the generation, transportation, and degradation of amyloid-β protein (Aβ) oligomers in Alzheimer’s disease (AD). EVs are thus increasingly recognized to play a neuroprotective role in AD, through their ability to counteract the neurotoxic effects of Aβ, possibly through interactions with specific receptors on cell membranes. Our previous studies have identified the amylin receptor (AMY), particularly AMY3 subtype, as a mediator of the deleterious actions of Aβ *in vitro* and *in vivo* experimental paradigms. In the present study, we demonstrate that AMY3 enriched EVs can bind soluble oligomers of Aß and protect N2a cells against toxic effects of this peptide. The effect was specific to amylin receptor as it was blocked in the presence of amylin receptor antagonist AC253. This notion was supported by reduced Aβ binding to EVs from AMY depleted mice compared to those from wild type (Wt) mice. Finally, application of AMY3, but not Wt derived, EVs to hippocampal brain slices improved Aβ-induced reduction of long-term potentiation, a cellular surrogate of memory. Collectively, our observations support the role of AMY receptors, particularly AMY3, in EVs as a potential therapeutic target for AD.

## Introduction

Alzheimer’s disease (AD) is the most common neurodegenerative disease that causes progressive loss of memory and other cognitive impairments. It is characterized, in part, by the accumulation of extracellular aggregates of beta amyloid (Aß) protein that is derived from the amyloid precursor protein (APP) through enzymatic cleavage by secretase enzymes, and the presence of neurofibrillary tangles (NFT) composed of the hyperphosphorylated tau protein [1]. The mechanisms whereby spatial and temporal spread of AD pathology occurs within the brain are not precisely understood, but several studies have now postulated that the transmission of toxic misfolded proteins between cells and brain regions may represent a critical factor in disease progression and pathogenesis of AD [2–4].

Extracellular vesicles (EVs) are small cytoplasm containing sacs enclosed by a lipid bilayer and range in size from 30 to 10,000 nm in diameter. They are produced by various cells including neurons, and released into the extracellular fluid [5, 6]. They are recognized as essential tools for cell-to-cell communication, discarding by-products and unwanted proteins, and the transfer of important cargos (proteins, RNAs, and lipids) between the cells [5, 6]. Several studies have identified a role for EVs in the enrichment or generation, transportation, and degradation of Aβ oligomers [7–10]. In AD brains, EV markers, such as Alix, have been localized around amyloid plaques [8, 9]. There is accumulating evidence that EVs may serve a neuroprotective function in AD, in part, through their ability to counteract the deleterious effects of Aβ, possibly through specific receptors. Exosomes (smaller type of EVs) derived from N2a cells attenuated the ability of synthetic and AD brain derived Aβ to disrupt synaptic plasticity and impairment of long-term potentiation *in vivo* [11]. These effects were attributed to the sequestration of Aβ oligomers via exosomal surface proteins, such as the p75 neurotrophin receptor, which has a high binding affinity for Aβ oligomers [11]. In another study, Yuyama *et al*. showed that intracerebrally administered exosomes in mice act as potent Aβ scavengers by binding to Aβ through enriched glycans on glycosphingolipids on the exosome surface thereby facilitating Aβ clearance from the brain [12]. Aβ oligomers bind strongly to the ganglioside, GM1, located on cell surface membranes but blocking the sialic acid residue on GM1 decreased Aβ oligomer-mediated long term potentiation (LTP) impairment in mouse hippocampal slices [13]. Collectively, these data suggest that EVs can interact with extracellular Aβ species through specific surface receptors that can help Aβ clearance thereby ameliorating its toxic effects and potentially providing a novel therapeutic intervention for AD.

Our group and other research groups have highlighted the role of amylin receptor (AMY) as a putative receptor for the expression of the deleterious effects ascribed to Aβ in the context of AD [14–18]. Amylin receptor is a Class B G-protein-coupled receptor comprised of heterodimers of calcitonin receptor (CTR) and one of three receptor activity modifying proteins (RAMP1-3) that generate multiple subtypes of amylin receptors, AMY1-3 [19]. We have shown that amylin receptor antagonists such as AC253 peptide and its cyclic analogue block the effect of Aβ toxicity *in vitro*, and that systemic administration of such antagonists improves memory and aspects of AD pathology including amyloid plaque burden and neuroinflammation [20]. Furthermore, genetic depletion of amylin receptors in transgenic AD mouse models results in an improvement in spatial memory and attenuation of some aspects of AD brain pathology [18].

In the current study, we hypothesized that binding of soluble oligomers of Aß to AMY3 containing EVs attenuates the toxicity normally observed upon exposing neuroblastoma N2a cell cultures to Aβ. EVs were generated from either subtypes of AMY receptor-expressing or wild-type (Wt) HEK293 cells and tested for their cytoprotective effects of such EVs in N2a cells exposed to soluble oligomeric Aß. We demonstrate that EVs generated from AMY expressing cells, particularly the AMY3 receptor subtype, protect N2a cells against soluble oligomeric Aß exposure. Furthermore, application of AMY3, but not Wt, derived EVs to mouse hippocampal brain slices improved hippocampal long-term potentiation, a cellular surrogate of memory. Finally, EVs generated from brains of AMY depleted mice demonstrate less Aß binding than those obtained from Wt mice. Our findings provide evidence for targeting AMY receptors as a potential therapeutic target in AD.

## Materials and methods

All experiments were carried out with the approval of the Animal Care Use Committee of the Health Research Ethics Board at the University of Alberta (Protocol AUP 00000268) and in accordance with guidelines set by the Canadian Council for Animal Care regarding humane and ethical treatment of animals.

### Cell lines

HEK AMY3 transfected GFP-positive Human Embryonic Kidney 293 cells that express AMY3 receptor (CTR+RAMP3) as previously reported were used [14]. For controls, wild type Wt GFP-positive HEK293 cells were used. Cells were cultured in DMEM (Invitrogen) with 10% exosome depleted FBS (Thermofisher) and grown at 37°C, 5% CO_2._ N2a mouse neuroblastoma cells (Invitrogen) were grown in DMEM with 5% FBS (Invitrogen).

### Drugs

Oligomeric Aβ_1–42_ was prepared according to published protocol [20]. Briefly, Aβ_1–42_ (rPeptide) was dissolved to 1 mM in 100% hexafluoroisopropanol, that was subsequently removed under vacuum, and the peptide was stored at -20°C. For oligomeric conditions, the peptide was first re-suspended in DMSO to 5 mM, then water added to bring it to a final concentration of 1 mM, and the peptide incubated at 4°C for 24 h. AC253 was purchased from American Peptide (Sunnyvale, CA) and dissolved to 1 mM in water and kept at -80°C. Aliquots of Aβ and AC253 were further diluted to final application concentration with cell culture medium.

### EVs isolation

HEK AMY3, and HEK Wt cells were grown in DMEM with 10% exosome depleted FBS (Thermofisher,A2720801), and 1% Penicillin/Streptomycin under a humidified environment of 5% CO_2_ incubator at 37°C. EVs were isolated as previously described with some modification. In brief, each batch a total of 4 culture dishes (20 × 10^6^ cells/dish) per condition were necessary for EV preparations from each cell type [21]. EVs containing media was fractionated by centrifugation at 1,500× *g* for 10 min at 4°C to remove cellular debris, then followed by centrifugation at 10,000× *g* for 30 min then, finally the supernatants were then filtered through 0.22 μM filter and then ultracentrifuge at (100,000 g × 1 h) on a Ti70 rotor (Beckman Coulter). The final pellet was then resuspended in PBS 1 ml. The amount of EVs used was expressed in terms of total protein which was determined using the Pierce BCA assay kit (Thermo Scientific). They were then characterized using a combination of Western blot, electron microscopy and dynamic light scattering (DLS).

### Western blot

Isolated EVs were characterized by immunoblot. Denaturing and reducing conditions (0.5% SDS, 25 mM DTT) were used to solubilize proteins prior to electrophoresis to detect targeted proteins. Proteins were transferred to nitrocellulose membrane using standard procedures, and then blocked with LI-COR blocking buffer. Blots were further incubated with primary antibodies overnight at 4°C on a shaker. Primary antibody used for 6E10 for Aß (biolegend, 803001), anti-Hsp70 antibody (Abcam, ab2787), anti-ALIX antibody (Abcam, ab117600), Anti-TSG101 antibody (Abcam, ab30871). IRDye 800CW goat anti-rabbit and IRDye 680CW goat anti-mouse (LI-COR, 1:10,000) were used as secondary antibodies. Blots were imaged using LiCor Odyssey CLX imaging system.

### Electron microscopy

5 μl drops of EVs (50 μg/ml) were loaded onto carbon-coated 200 μm copper grids and incubated for 1 min. The samples were then stained with 2% uranyl acetate for 2 min, and excess solution carefully removed, and the grid left to air dry for 24 hours. Images were captured using an electron microscope (JEM-2100) operated at 100 kV.

#### Dynamic light scattering (DLS)

The size distribution of exosome samples was carried out with a Malvern Zetasizer-Nano Instrument. A He-Ne laser with wavelength 632 nm was used to detect backscattered light at a fixed angle of 173°. The software (DTS v6.20) provides both the mean size and polydispersity by cumulants analysis. The solution viscosity and refractive index (1.33) were assumed to be of water for calculation purposes. Data were collected using a 10mm quartz cuvette filled with 150μL samples. The data were collected without attenuation and a minimum number of 10 consecutive runs of 10sec each was averaged to obtain the autocorrelation function. Particle size was calculated by the manufacturer’s software through the Stokes–Einstein equation assuming spherical shapes of the particles [22].

### Fluorescence microscopy

EVs were labelled with 1,1′-dioctadecyl-3,3,3′,3′-tetramethylindocarbocyanine (DiI) dye (Sigma-Aldrich), according to the manufacturer’s protocol. Briefly, 4 μL DiI dye was mixed with exosome suspension (100μg/ml) in diluent C and incubated for 10 min at 37°C. The labelling reaction was stopped by adding 20 ml chilled PBS. Labelled EVs were ultra-centrifuged at 100,000×*g* for 70 min, washed with PBS, ultra-centrifuged again at 100,000×*g* and the pellet was resuspended in PBS and stored at −80°C for further experiments. To assess the cell uptake of the EVs, N2a cells were seeded on coverslips in 12-well plates at a density of 1.5 × 10^5^ cells/well until 50% confluence was achieved and subsequently incubated with EVs labelled with DiI (0.71 ± 0.33 μg/μl from conditioned media) diluted in culture medium at 37°C for 6 h. In order to use equal amounts of EVs in the cell cultures, EVs protein content was quantitated by using BCA. Subsequently, cells were washed three times with PBS, fixed in 4% paraformaldehyde in PBS for 10 min, and mounted in DAPI mounting media. The cells were imaged using a Zeiss Axioplan-2 microscope (Carl Zeiss Microscope Systems, Toronto, ON, Canada) and AxioVision software (version 4.8) with identical photo settings.

### Cell viability assay

Cell viability was measured with the 3-(4,5-dimethylthiazol-2-yl)-2,5-diphenyltetrazolium bromide (MTT) colorimetric assay as previously described [20]. N2a mouse neuroblastoma cells were used. Cells were seeded to 5000 cells/well in a 96-well plate in DMEM-5% FBS for overnight. Cells in culture were treated in serum free media (to prevent enzymatic degradation of AC253 peptide) for different treatments: EVs (100 μg ml^−1^) only, EVs with Aß (10 μM), AC253 (10 μM) preincubation of cells for 8 h followed by addition of EVs and Aß for 48 hr. After MTT exposure for 3 h, cells were incubated in isopropanol with 10% Triton X-100, and the solubilized formazan was measured by spectrophotometry using the SOFTmax Pro microplate reader (Molecular Devices). Absorbance was measured at λ = 570 nm.

### Dot blot

Dot blot was carried out as follows: Nitrocellulose Membrane was first soaked with PBS till thoroughly saturated. The membranes were then incubated with samples in triplicates for 30 mins, which was followed by washing three successive times with PBS, then air dried. Membranes were then blocked using Odyssey blocking buffer (LI-COR), following which a second incubation was performed with a primary antibody (either for Aß (6E10, Biolegend, 803001), CTR (Abcam, ab11042), or RAMP3 (Abcam, ab56684)) overnight. The membranes were then washed, treated with secondary antibody, and visualized using Odyssey CLX image system (LI-COR). Bands were quantified using Image Studio Lite software (LI-COR, USA).

For EVs binding to Aß, 1 μg of Aß oligomers was added to identical volumes of EVs (160 μg/200μl) and incubated at 37°C for 30 min in 10 ml PBS. Thereafter, EVs were separated from the unbound Aβ by centrifuging for 1 h at 100,000 g. The exosome pellet was dissolved in 2× sample buffer and 25% of the mixture used for dot blot to detect amount of exosome-bound Aβ using 6E10 antibody followed by secondary fluorescently labelled antibody. Or the Aβ42 levels that bind EVs after 30 mins incubation were measured separately by commercial ELISA kits (Thermo Fisher Scientific) for Aβ42 levels.

### Aβ aggregates deposition inhibition

N2a cells were seeded at 5000 cells/well in a 96-well plate. After culturing for 24 h, cells were either treated with 10μM fluorescent Aβ_1–42_ HiLyte Fluor 555 only (AnaSpec Inc. Fremont, CA), or combination of the labelled Aß and EVs in culture medium for 6 hours then washed. Cells were fixed with 4% paraformaldehyde for 20 min, permeabilized with 0.2% Triton X-100 in PBS, blocked with Odyssey blocking buffer (LI-COR) and visualized using fluorescence microscopy EVOS M5000 (Thermo Fisher Scientific).

For quantitative measurement of aggregates in cells, the fluorescent density was quantified in a 96 well plate using a plate reader (Varioskan Lux, Thermofisher Scientific) at Excitation 550, Emission 566.

### Immunoprecipitation

Presence of the amylin receptor on the surface of EVs was detected using immunoprecipitation Kit (Dynabeads Protein G, Invitrogen) following the manufacturer protocol. Briefly, 50 μl Dynabeads was transferred to a tube, and resuspended in 200 μl antibody binding and washing buffer containing 10 μg CTR antibody, and incubated for 10 minutes with rotation. The Dynabeads-CTRantibody complex was then washed and incubated with AMY3 EVs (100 μl from stock 100μg/ml) for 10 minutes with rotation. Finally, the bound EVs were eluted with 20 μl Elution Buffer, and their identity confirmed with TSG101 antibody using western blot. Another set of Dynabeads treated with Wt EVs was run as control.

### Animal models

Mice were housed individually under standard laboratory conditions (1212 h light/dark cycle, lights on at 0600 h) with a room temperature of 21°C. Water and food were available *ad libitum* unless otherwise indicated. EVs were extracted from either male or female 6month-old wild type (C57BL/6 background) or heterozygous CTR (HetCTR) mice (same background as wild type). HetCTR mice generated breeding pairs obtained from Drs. R.A. Davey and JD Zajac and have been shown to express 50% reduction of CTR [18, 23]. For *in vitro* LTP experiments, we used FVB strain of mice.

### Exosome purification ex vivo

Isolation of brain EVs from extracellular space of fresh mice brains tissues was performed as previously described [24, 25]. The dissected tissue was dissociated with papain 15 min at 37°C (20 units/ml, Sigma-Aldrich) followed by filtration through a 0.2 μm syringe filter (Thermo Scientific) to separate extracellular matrix from cells. 5 mice brains were used for each group. The crude EVs were then isolated by differential centrifugation method as previously described *in vitro*. Then the centrifuged pellet was resuspended in PBS for further experiments. Characterization of the EV was done using western blot and electron microscopy as mentioned above. CTR detection and Aß binding was performed with dot blot.

### Electrophysiology

Brains were quickly removed from FVB strain of mice following decapitation, placed in a cold artificial cerebral spinal fluid (aCSF) on a vibratome chamber and transverse section cut through the hippocampus. The aCSF contained (in mM) 124 NaCl, 3 KCl, 2.4 CaCl2, 2 MgCl2, 1.25 NaH2PO4, 26 NaHCO3 and 10 D-glucose, and was equilibrated with 95% O2 and 5% CO2. Hippocampal slices (400 μm thick) were maintained in aCSF-filled holding chamber at room temperature for at least 1 h and individually transferred to the submerged glass bottom recording chamber, which was constantly perfused with aCSF (2 ml/min) at 30°C. Field excitatory postsynaptic potential (fEPSP) was recorded with a metallic (Pt/Ir) electrode (FHC, Bowdoin, ME) from the stratum radiatum layer of Cornu Ammonis 1 region of the hippocampus (CA1) area, and the Schaffer collateral afferents were stimulated with 100-μs test pulses via a bipolar cluster electrode (FHC) [26]. For long-term potentiation (LTP) experiments, the stimulus strength was set to elicit 40–50% of the maximum fEPSP amplitude and test pulses were delivered to Schaffer collaterals once every 30 seconds. LTP was induced by 3-theta-burst stimulation (3-TBS) protocol (each burst consisted of 4 pulses at 100 Hz with a 200-ms interburst interval). Before applying the solution containing EVs, the responses were monitored for at least 10 min to ensure a stable baseline of fEPSP. To determine whether the magnitude of LTP differed significantly between groups, average responses during the last 20-min block of recordings (40–60 min after TBS) were compared.

### Statistical analysis

Statistical Analysis—Values are means ± SE. Significance was determined using either student t test, or one-way analysis of variance (ANOVA), followed by Tukey’s test when appropriate, with Prism software (GraphPad Prism 5, GraphPad Software, San Diego, CA). p < 0.05 was considered significant.

## Results

### EVs isolation and characterization

**Fig 1:**
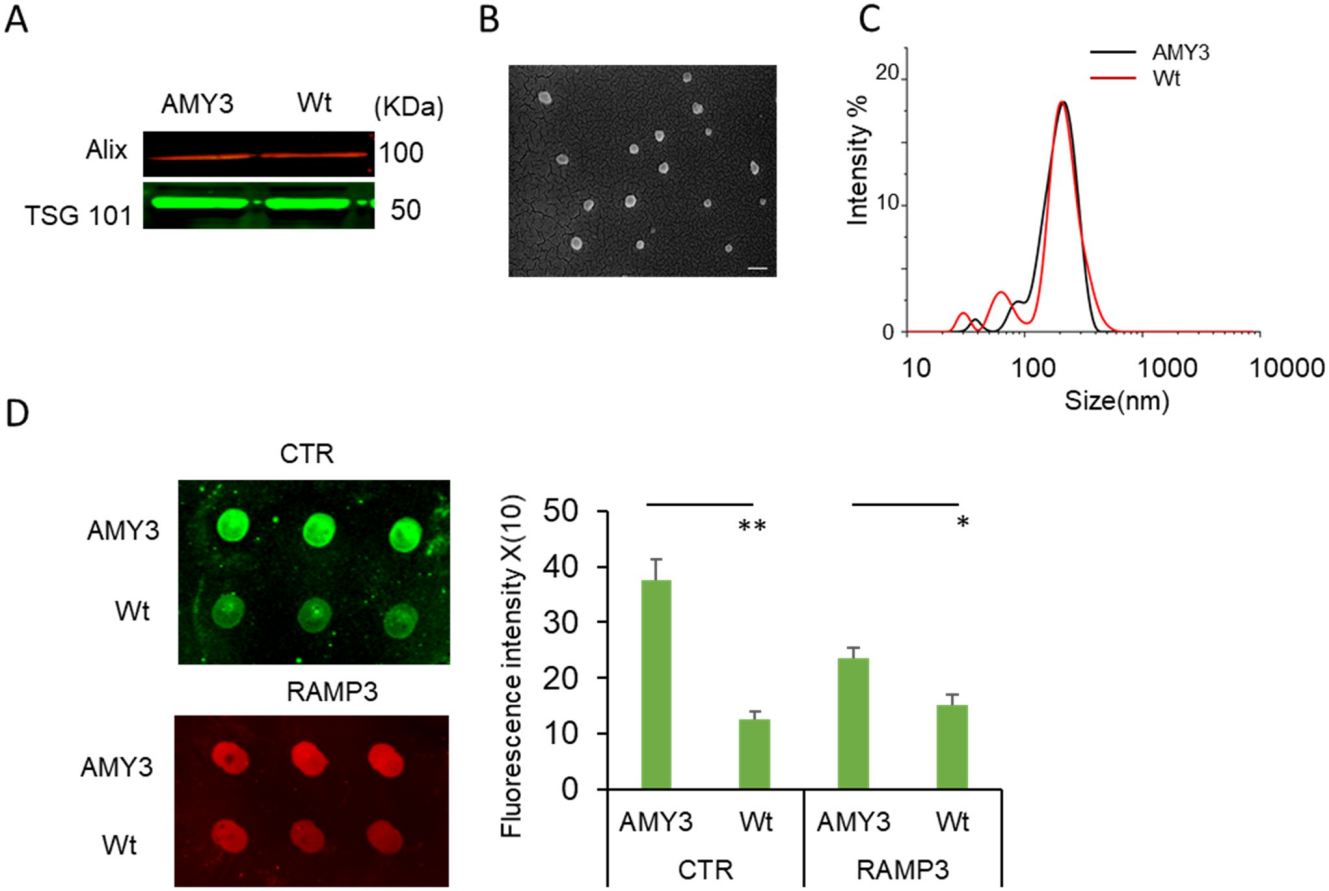
Characterization of extracellular vesicles (EVs) from HEK AMY3 and HEK Wt cells. **A**, Western blot detection of the Alix and TSG101, two commonly used EV markers. Molecular weights are presented in kDa. **B**, Transmission electron microscopy of EVs derived from AMY3 cells using a negative staining method. EVs appear as closed vesicles (Scale bar = 200 nm). **C**, Dynamic light scattering (DLS) showing the relative size of HEKAMY3 and Wt EVs. **D**. Dot blots (in triplicate from each EV preparation) show amylin receptor heterodimeric components CTR (green) and RAMP3 (red) proteins are more abundant in EVs derived from HEK-AMY3 than HEK Wt cells. Histogram showing the relative quantification of CTR and RAMP3intensity (from dot blots) in AMY3 compared to Wt EVs. Data are presented as mean ± SEM, n = 3, and an average from three independent EV preparations, ** p<0.01, * p<0.05).

In the current experiment, we isolated EVs from HEK AMY3 cells that are transfected to express the AMY3 receptor subtype (heterodimeric combination of CTR and RAMP3) [14]. EVs were collected from culture supernatants of HEK AMY3 cells or HEK Wt cells (as control) using sequential centrifugation. The EVs isolated from HEK AMY3 cells and HEK Wt cells were identified by detection of EVs markers Alix and TSG101, that are known to characterize EVs, using western blot (Fig 1A). AMY3 EVs morphology was further confirmed by electron microscopy, which demonstrated small membrane vesicles 30–150 nm in diameter (Fig 1B). We also independently measured the size of EVs generated from Wt and AMY3 cells using dynamic light scattering (DLS) method. The size distribution of EVs is shown in (Fig 1C) and the average diameter of 150nm is consistent with that reported by others [27, 28] and our electron microscopic data. We next determined the level of AMY receptor expression in EVs using dot blot analysis. A significantly increased expression of CTR and RAMP3 proteins the 2 heterodimeric components of amylin receptor was observed in AMY3 EVs compared to those derived from Wt cells EVs (Fig 1D).

### AMY3 EVs are cytoprotective against Aß toxicity

**Fig 2:**
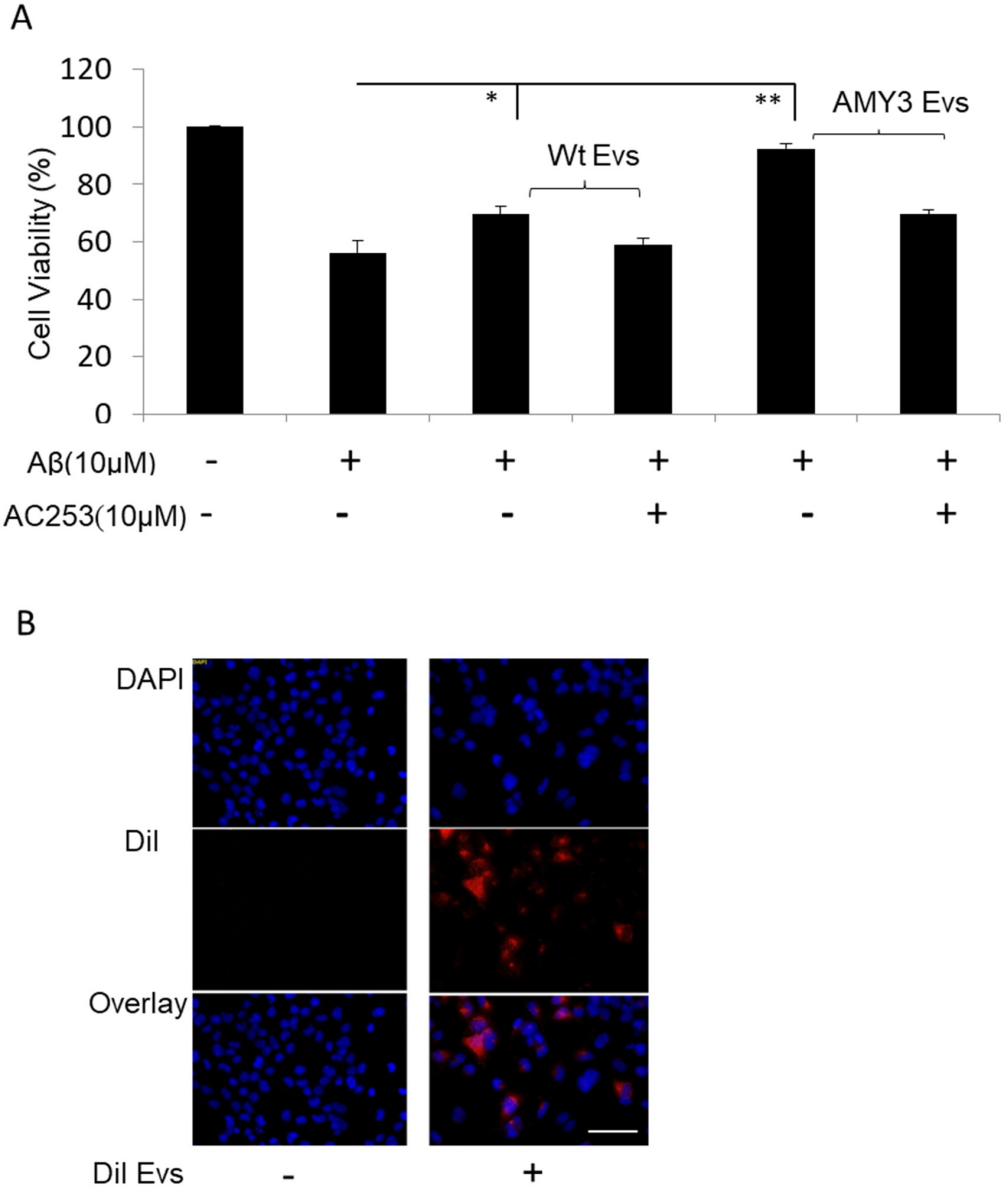
EVs from AMY3 expressing cells confer cytoprotection against Aß toxicity. **A**, MTT assay for cell viability showing that EVs derived from HEK AMY3, but not HEK Wt cells, protect against Aß-induced cell death in N2a cell cultures after 48 h incubation. This effect was reduced with pre-treatment of cell cultures with the amylin receptor antagonist, AC253 for 24 h (data presented as ± SEM, n = 3 samples/group, experiment was repeated in triplicate, * p < 0.05; ** p < 0.01). **B**, Fluorescence microscopic images showing the presence of DiI labeled EVs (red) within N2a cells following 2 h of incubation (panels on the right). Controlled N2a cells not exposed to labeled DiI EVs are shown in panels on the left. Scale bar = 100 μm.

We next examined the *in vitro* effects of EVs derived from AMY3 overexpressing cells on N2a cells in the presence of soluble oligomeric Aß_1-42_. We co-incubated N2a cells with EVs and Aß for 48 hours in serum free media, then measured cell viability using the MTT assay. Aß-induced cytotoxicity was significantly reduced following co-incubation with EVs derived from AMY3 cells compared to those from Wt cells (cell viability of N2a cells in presence of Aß = 55± 4%, Aß + AMY3EVs = 92± 1%, Aß + Wt EVs = 69± 2%) (Fig 2A). These cytoprotective effects were significantly reduced when the AMY3 EVs were pretreated with the amylin receptor antagonist, AC253, prior to co-incubation with Aß (Fig 2A). In order to determine whether our cytoprotective effects against Aß toxicity are specific to EVs derived from AMY3 receptor subtype, we also examined the effects of EVs generated from AMY1 and AMY2 expressing HEK cells (S1A Fig) using the same experimental paradigm and MTT assay. Our data indicate that EVs derived from AMY3 HEK cells demonstrate significantly greater cytoprotection against Aß compared to those from AMY1 or AMY2 expressing cells (cell viability of N2a cells in presence of Aß = 55 ±5%, Aß + AMY3 EVs = 91 ± 4%, Aß + AMY1 EVs = 68 ± 5%, Aß + AMY2 EVs = 59%± 4) Pretreatment with AC253 reduced the cytoprotective activity of AMY3 EVs (S1B Fig). Finally, we examined the EV binding and internalization into N2a cells using fluorescence microscopy. Microscopic images revealed binding of DiI fluorescently labeled EVs within N2a cells (Fig 2B).

### EVs binding to Aß in vitro and effects on Aß cells aggregates deposition

**Fig 3:**
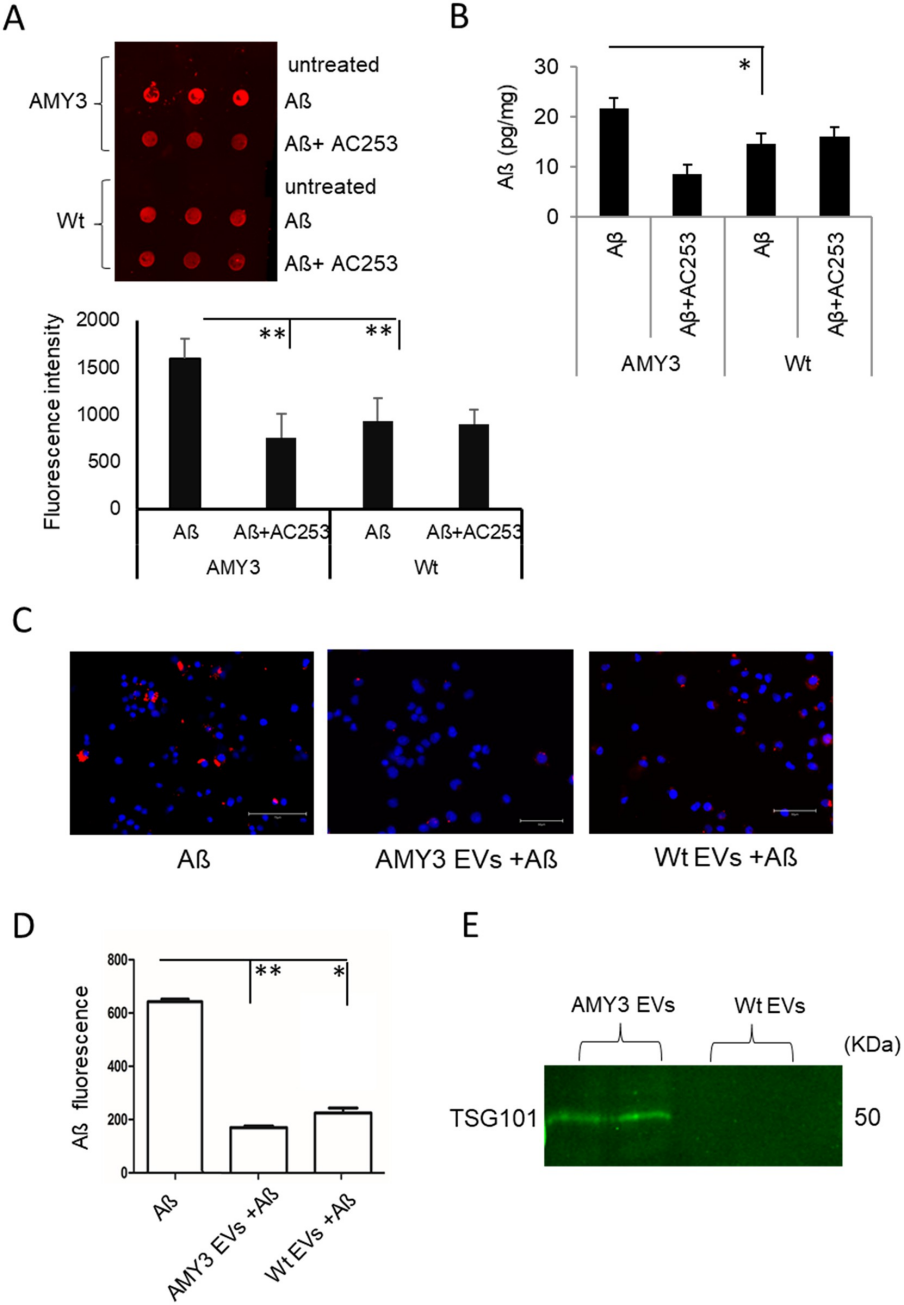
HEK AMY3 derived EVs bind to Aß though AMY receptors expressed on N2a cells *in vitro*. **A**, Dot blots (top panel) showing EVs generated from AMY3 cells bind Aß to a greater degree than EVs from Wt cells. Preincubation with the amylin receptor antagonist, AC253, blocks Aß binding to EVs derived from AMY3, but not those from Wt cells. Aβ levels in the EVs were measured from dot blots with an Aβ antibody (6E10) and a fluorescently labeled secondary antibody. EVs not treated with Aß were used as controls. Histograms (lower panel) showing quantification of mean fluorescence of Aβ in EVs (Data are ± SEM, n = 3 samples/group, experiment was repeated in triplicate; ^**^p< 0.01). **B**, ELISA quantification of Aβ binding to AMY3 or Wt EVs with and without pre-incubation with AC253. Aβ level was normalized to the total protein content in the measured samples. Data is presented as mean ± SEM, n = 3 per group, *p< 0.05. **C**, Effect of AMY3 EVs on Aß amyloid accumulation in N2a cells. Fluorescently labeled Aß (red) incubated with N2a cells (Dapi stained the nuclei in blue) results in accumulation of amyloid aggregates around or within cells. The Aß accumulation can be attenuated in the presence of EVs generated from either AMY3 or Wt cells. **D**, Histograms showing the quantification of Aß labelled profiles in the presence of AMY3 or Wt EVs and without EVs. **E**, Using a immunoprecipitation, AMY3- and Wt- EVs were separately eluted from Dynabeads prepared with calcitonin receptor (CTR) antibody and detected for exosome-specific protein, TSG101, using Western blot.

In order to address the possible mechanisms underlying the protective action of EVs against Aβ induced neurotoxicity, we examined the ability of EVs to bind and sequester Aß *in vitro*. EVs were incubated with Aß and Aß levels in EVs were then evaluated using Dot blot. Data from Dot blots showed that AMY3 derived EVs bind more Aß than Wt EVs, and this Aß binding is decreased when the EVs are preincubated with the amylin receptor antagonist, AC253 (Fig 3A). As the 6E10 antibody can bind to APP and Aß oligomers, we used untreated EVs as negative controls. Untreated EVs did not show any signal with 6E10 antibody, which confirms that the fluorescent signal we observed is specific to Aß binding. ELISA quantification of Aβ oligomers further confirmed greater binding affinity of AMY3 EVs for Aß than Wt EVs (AMY3 EVs bind 22±3 pg/mg exosomal Aß protein compared to 13±2.3 pg/mg for Wt EVs). The Aß binding was decreased when the AMY3 EVs were pretreated with AC253, but not with Wt EVs (Fig 3B).

Next, we assessed the effect of the AMY3 EVs on amyloid pathology in N2a cells. Exposure of N2a cells to soluble oligomeric fluorescently labeled Aβ results in formation of amyloid deposits as shown with fluorescence microscopy, which can be attenuated in the presence of EVs generated from either AMY3 or Wt cells (Fig 3C). Quantification of amyloid deposits in N2a cell cultures reveals a significant decrease when either AMY3 or Wt EVs were pre-incubated with fluorescent Aβ (Fig 3D). To confirm the presence of CTR on the surface of EVs and its binding specificity, immunoprecipitation with CTR antibody was performed. CTR immobilized beads were used to capture AMY3 EVs, which were then eluted and their identity confirmed using western blot that demonstrated presence of the EV marker, TSG101. On the other hand, EVs generated from Wt cells did not bind to the CTR antibody at a level that could be detected on western blot (Fig 3E).

### Effects of AMY3 EVs on Aβ-induced depression of hippocampal long term potentiation (LTP)

**Fig 4:**
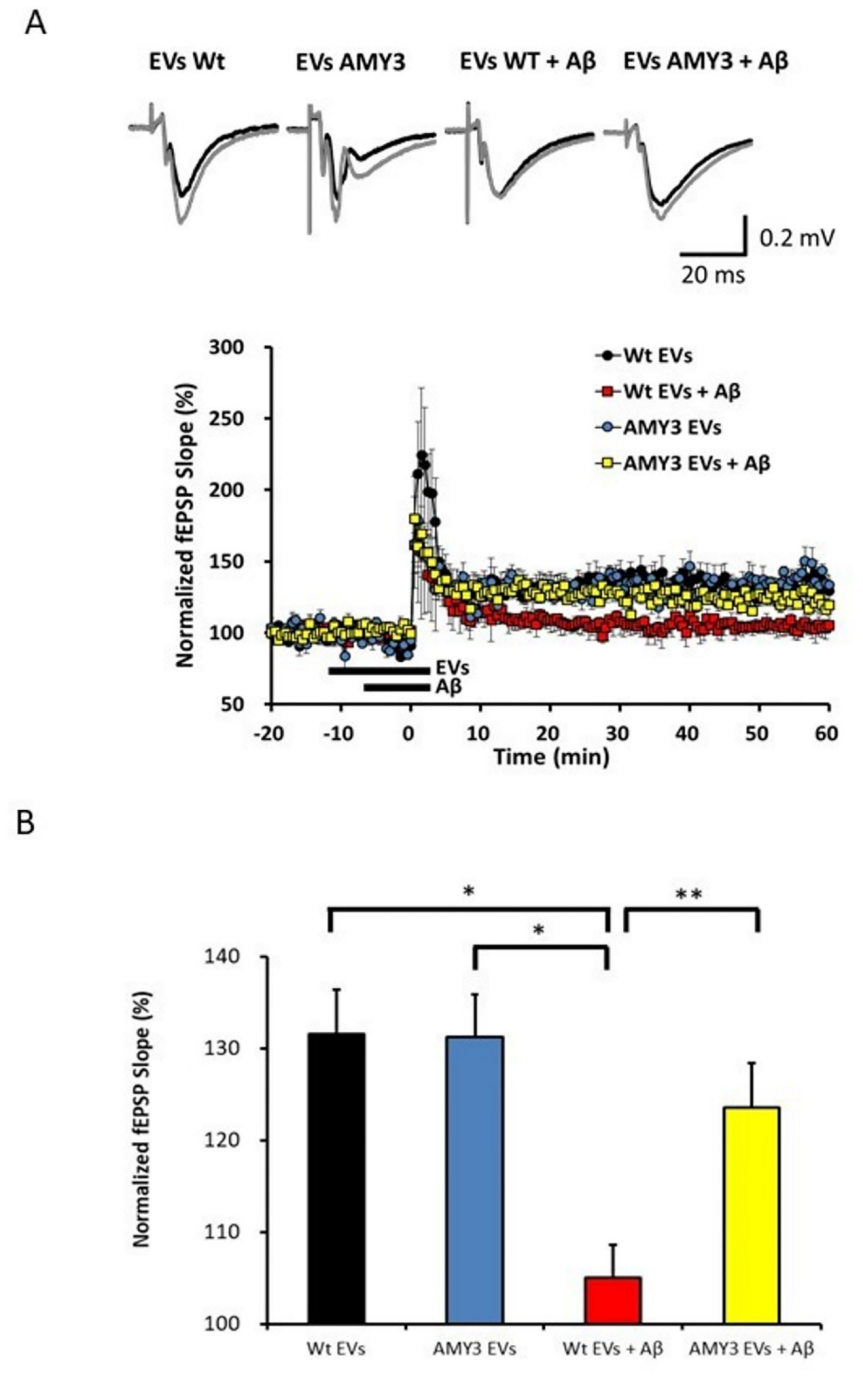
AMY3 derived EVs attenuate Aβ-induced depression of hippocampal LTP. **A**, LTP in mouse hippocampal brain slice was induced by ×3 TBS protocols and EVs from either AMY3 or Wt cells were perfused 5 min prior to applications of soluble oligomeric 50 nM Aβ_1–42._ Each point indicates the fEPSP slope normalized to the average baseline response over 10 min prior to EV applications; the bars indicate onset and duration of application (Wt EVs (black), Wt EVs + Aβ_1–42_ (red), AMY3 EVs (blue), and AMY3 EVs + Aβ_1–42_ (yellow), n = 6 for each group, one brain slice per mouse). All data are presented as mean ± SEM. Traces shown above the LTP data are the average of fEPSPs recorded during baseline (black) and 40–60 min after ×3 TBS (grey). Calibration: 0.2 mV, 20 ms. **B**, Summary bar graphs show significant reductions in LTP after application of Wt EVs with 50 nM Aβ_1–42_, but not AMY3 EVs with 50 nM Aβ_1–42_ *p < 0.05, ** p<0.01, ANOVA followed Tukey’s post hoc test).

Applications of EVs generated from Wt and AMY3 expressing cells alone did not result in a significant alteration of LTP in Wt mice at hippocampal Schaffer collateral-CA1 synapses (Fig 4A and 4B). Exposure of the hippocampal slices to soluble oligomeric Aβ_1–42_ (50nM) in presence of Wt EVs significantly depressed the LTP induced by application of 3-TBS protocol at the CA1 region. However, in presence of AMY3 EVs, the Aβ_1–42_ -induced reduction in LTP was partially restored to the levels closer to those observed following applications of Wt and AMY3 EVs without Aβ (n = 6 for each group, one brain slice per mouse, *p < 0.05 for Wt EVs vs Wt EVs + Aβ_1–42_ and AMY3 EVs vs Wt EVs + Aβ_1–42_, **p < 0.01 for Wt EVs + Aβ_1–42_ vs AMY3 EVs + Aβ_1–42_, ANOVA followed Tukey’s post hoc test). These results indicate that Aβ-induced impairment of hippocampal LTP was partially rescued by presence of EVs derived from AMY3 expressing cells, but not EVs derived from Wt cells.

### Aβ binding to EVs derived from mice with genetically depleted amylin receptors

**Fig 5:**
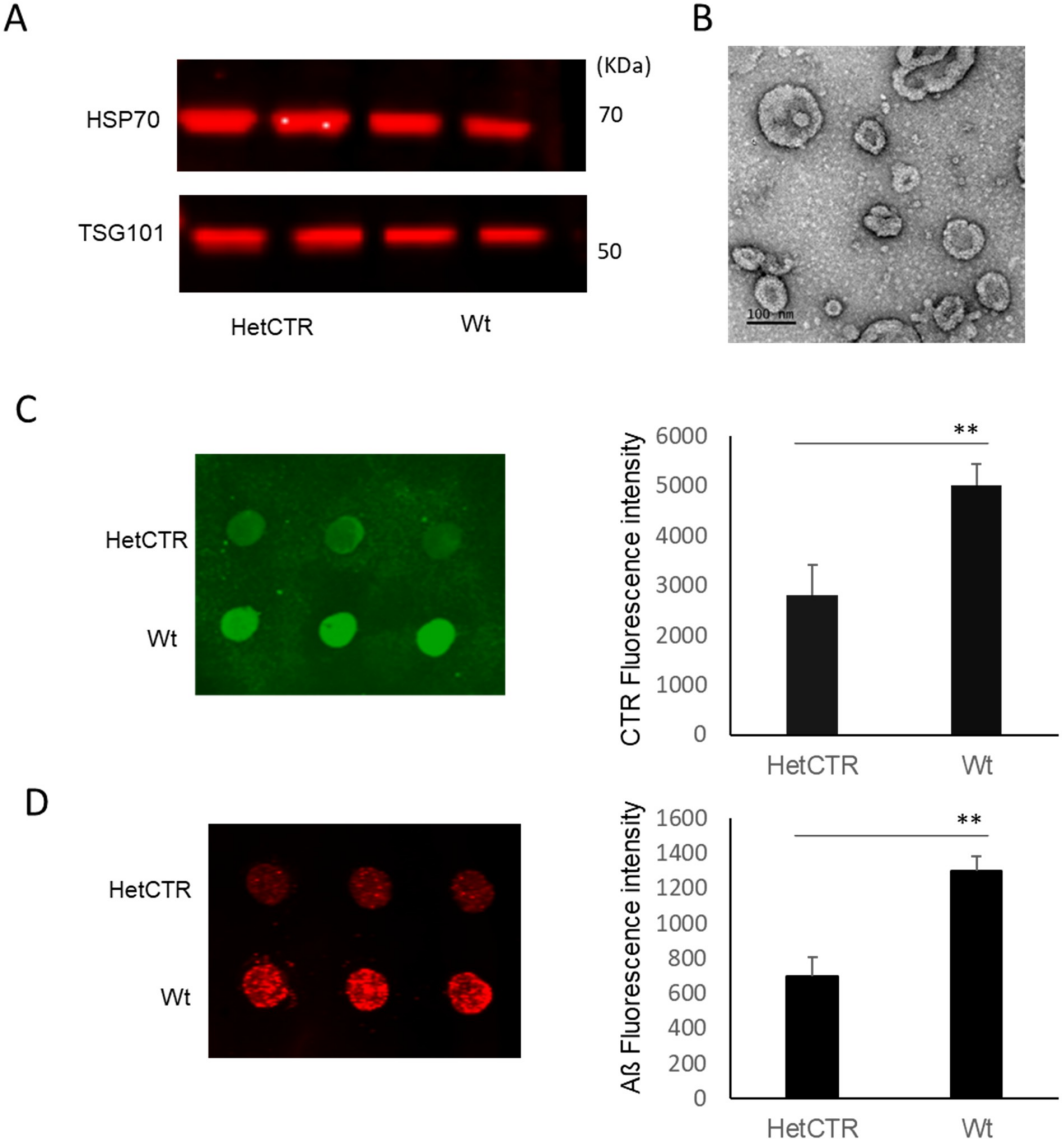
EVs from heterozygous AMY/calcitonin receptor (HetCTR) depleted mice and Aβ binding. **A**, Characterization of EVs from age-matched HetCTR mice brains and Wt mice using Western blot detection of EV markers, Hsp70, and TSG101. **B**, Transmission electron microscopy of EVs from brain of HetCTR mouse brain using a negative staining method. EVs appear as closed vesicles of 30–200 nm in diameter (Scale bar = 100 nm). **C**, Dot blot (left panel) showing greater abundance of CTR protein from EVs extracted from Wt mice compared to those from HetCTR mice. Histogram (right panel) showing the quantification of CTR from dot blots (Data presented as mean ± SEM, n = 5 mice each group. ** p<0.01. **D**, Dot blots (left) and histograms (right) demonstrating increased Aβ binding to EVs from Wt mice compared to HetCTR mice following 30 min incubation the peptide. (Aβ levels are presented as the mean ± SEM, n = 5 mice each group, ** p<0.01).

EVs generated from HetCTR mice, in which the CTR component is reduced by 50%, were initially identified and characterized using Western blot and electron microscopy (Fig 5A and 5B). Reduction of CTR expression in EVs generated from HetCTR mice compared to Wt age-matched control mice was confirmed using Dot blot analysis (Fig 5C). EVs derived from HetCTR mice when pre-incubated with fluorescent Aß demonstrated significantly less binding to the peptide compared to EVs from Wt mice (Fig 5D).

## Discussion

Extracellular Vesicles (EVs) represent a heterogeneous group of membrane-bound cytoplasm containing vesicles that are secreted from different cell types into the extracellular fluids and contain proteins, nucleic acids, RNA [29]. Current evidence suggests that EVs may be intimately involved in pathogenesis of neurodegenerative diseases and that these cell-derived structures also sometimes referred to as exosomes could be exploited to target cells for the effective delivery of drugs and other disease modifying molecules [30]. In this study, we show that EVs derived from HEK293 cells that stably express the AMY3 receptor are capable of attenuating Aβ-induced cytotoxicity *in vitro*. Furthermore, we demonstrate that such effects are specific to the AMY receptor as an antagonist for the amylin receptor (AC253) abolishes cytoprotective activity following applications of EVs. Although EVs derived from all subtypes of AMY receptors demonstrate the similar cytoprotective activity, however, the rank order for this property appears to be AMY3>AMY1>AMY2. This observation is consistent with prior data showing similar preferential human amylin and Aβ activation of AMY3 versus AMY1 or AMY2 receptors in stably transfected HEK 293 cells [31]. We also show that EVs derived from AMY3 cells are capable of improving Aβ-induced depression of hippocampal LTP further supporting AMY3- Aβ interaction.

Several mechanisms have been proposed to explain how EVs might exert their beneficial effects in a variety of *in vitro* and *in vivo* experimental paradigms. There is evidence that surface membranes of EVs express specific molecules or receptors which bind to extracellular Aβ and counteract the deleterious effects of this protein, thereby preventing Aβ-mediated neurodegeneration and synaptotoxicity [32]. Yuyama *et al*. demonstrated that mixing a preparation of seed-free soluble Aβ_1–42_ with EVs from primary neuronal cells caused significant increase in fibril formation [33]. The formation of such Aβ fibrils was accelerated through interaction with glycosphingolipid glycans present on surface of EVs. The EVs containing Aβ fibrils were subsequently internalized into microglia, transported to lysosomes and subsequently degraded and eliminated [33]. The *in vitro* observations were validated in another study by the same authors in an AD mouse model where injections of EVs (containing Aβ) into the hippocampus were co-isolated with microglial marker, Iba1 [12]. Furthermore, these authors also demonstrated that EVs derived from neuroblastoma cells when injected centrally into APP over-expressing mice resulted in reduced synaptotoxicity and Aβ levels. These beneficial effects were postulated to occur as a result of abundant glycosphingolipids contained within EVs that act as potential scavengers of Aβ and thus enhance its clearance from the brain [12]. Interestingly, EVs derived from astrocytes have been shown to promote aggregation of soluble Aβ species on the vesicle surface, a process that is critically dependent on sphingolipid ceramide [34]. In our study, we postulated that exosomes derived from AMY3 HEK cells express AMY3 receptors on their surface, which in turn bind soluble oligomeric Aβ and thus attenuate its cytotoxicity on N2a cell cultures. Two aspects of our *in vitro* experiments support this notion. First, pre-treatment of EVs with AC253, an AMY receptor antagonist, abolished the cytoprotective effects of the Evs against Aβ toxicity. Second, we performed immunoprecipitation experiments to detect the localization of amylin receptor on EVs. Western blots from the elute using the CTR antibody binding beads showed significantly higher number of EVs from AMY3 cells compared to those obtained from Wt cells. Thus, it appears that AMY3 receptors expressed on EV surface membrane bind to Aß oligomers thereby mitigating its toxicity. In order to further confirm the AMY receptor-Aß interactions, we utilized an *in vivo mouse* model where there is a genetic depletion of calcitonin receptor (CTR), a critical heterodimer comprising the AMY receptor [18]. Extracellular vesicles generated from brains of heterozygous CTR mice (50% reduction in CTR and hence AMY receptor deficient) demonstrated significantly less binding to Aß oligomers compared to those from Wt mice.

The beneficial effects of exosomes on synaptic plasticity have been previously reported in a study where intracerebroventricular infusion of either N2a or human cerebrospinal fluid derived exosomes attenuated the disruption of LTP induced by injection of soluble Aβ species in rats [11]. This effect is deemed to involve direct sequestration of synaptotoxic Aβ assemblies by EV surface proteins such as cellular prion protein, PrP^C^, that is known to bind to Aβ. Several studies have also examined the improvements in hippocampal LTP following traumatic brain injury in rats [35, 36], but to our knowledge, our study is the first to report an improvement in Aβ-induced reduction in hippocampal LTP in mouse brain slices following perfusion with AMY3, but not Wt, derived EVs. The rapid improvement in LTP within minutes of application of EVs would also seem to support a cell surface interaction between Aβ and AMY receptors on cell membranes of EVs.

In conclusion, we demonstrate that EVs derived from AMY3 expressing cells bind to Aβ oligomers and this interaction results in an attenuation of Aβ-induced cytotoxicity and depression of synaptic plasticity. These observations further support a role for AMY receptors, particularly AMY3, as a potential therapeutic target for AD.

## Supporting information

S1 Fig (DOC)

S1 Raw images (PDF)
