# Supplementary material for: Extracellular vesicles enriched with amylin receptor are cytoprotective against the Aß toxicity in vitro
Source: PLoS One. 2022 Apr 14;17(4):e0267164. doi: 10.1371/journal.pone.0267164 (PMC9009604; doi:10.1371/journal.pone.0267164)
Supplement: S1 Fig — (DOC) [file pone.0267164.s001.doc]

**Supporting information**

**
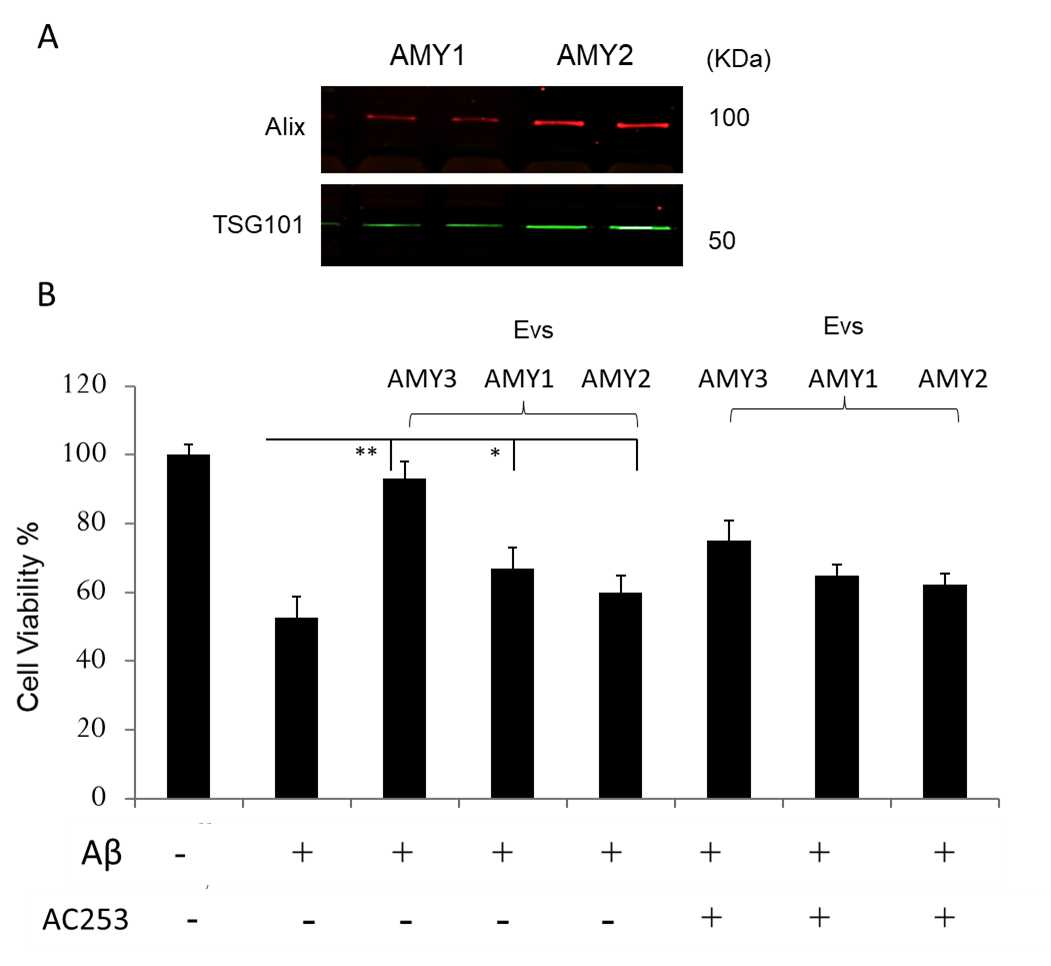
**

**Supplementary figure**

**Figure** **S1**: Differential neuroprotection conferred by EVs subtypes of AMY receptors against Aß toxicity. **A**, Western blots showing presence of EV markers, Alix and TSG 101, in EVs generated from AMY1 and AMY2 expressing HEK cells. **B**, MTT assay showing differential cytoprotection by EVs generated from AMY1-3 against Aß toxicity in N2a cell cultures (Data expressed as ± SEM, n = 3 samples/group, experiment was repeated three times, p < 0.05; **, p < 0.01).
