# Supplementary material for: Extracellular vesicles enriched with amylin receptor are cytoprotective against the Aß toxicity in vitro
Source: PLoS One. 2022 Apr 14;17(4):e0267164. doi: 10.1371/journal.pone.0267164 (PMC9009604; doi:10.1371/journal.pone.0267164)
Supplement: S1 Raw images — (PDF) [file pone.0267164.s002.pdf]

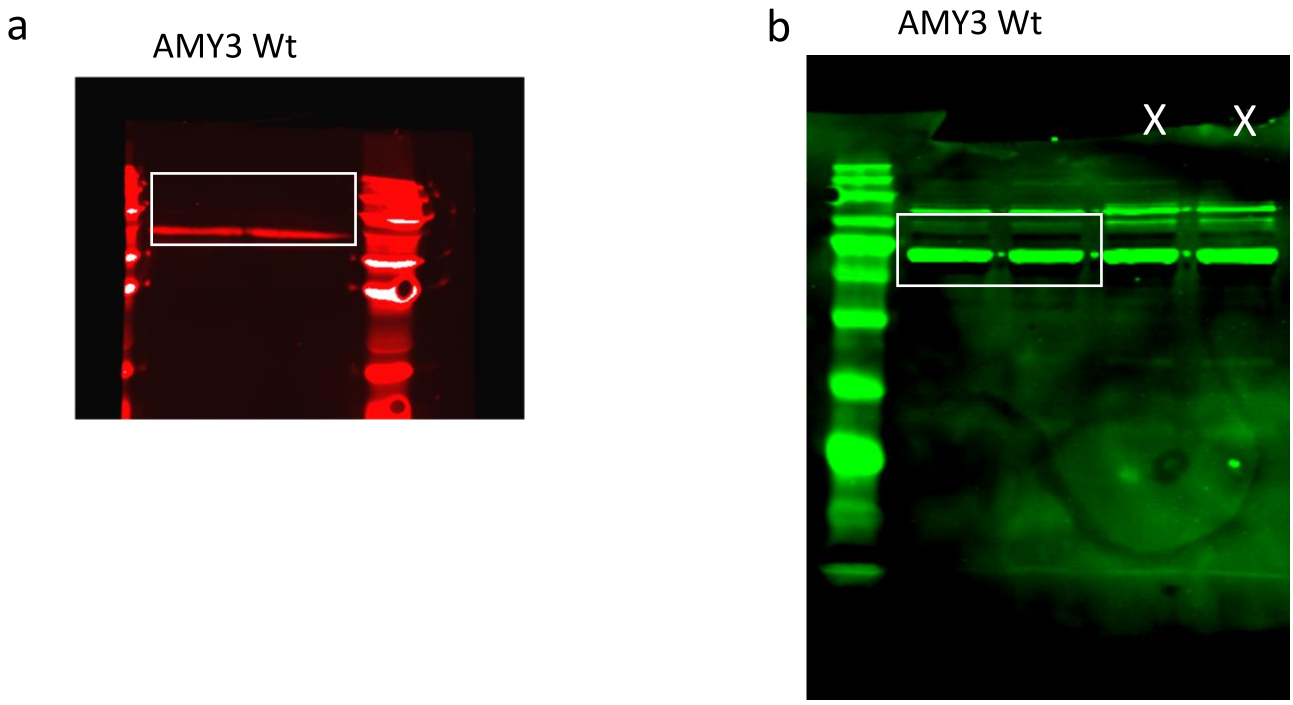

Raw images for western blot in Fig 1A, the EVs used in this data are from HEK AMY3 and HEK Wild type (Wt) cells. The antibodies used for a, and b are Alix and TSG101, respectively. LI-COR Odyssey imaging system was used to capture the image

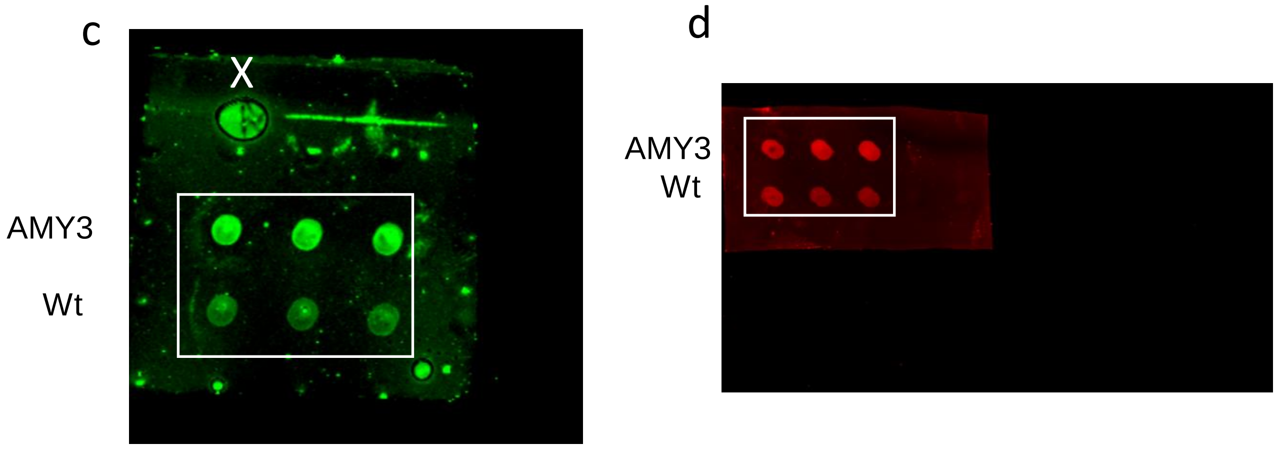

Raw images for dot blot in Fig 1D, the EVs used in this data are from HEK AMY3 and HEK Wt cells. The antibodies used for c, and d are CTR and RAMP3, respectively. LI-COR Odyssey imaging system was used to capture the image

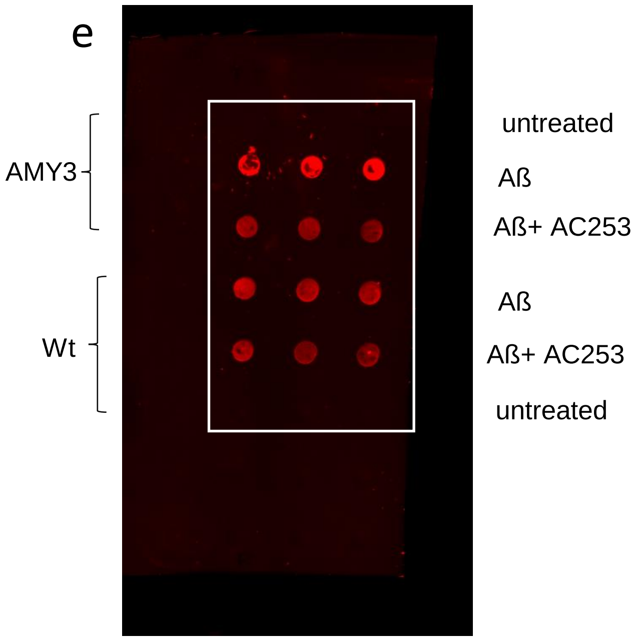

Raw image for dot blot in Fig 3A, the EVs used in this data are from HEKAMY3 and HEK Wt cells. The antibody used in e is 6E10. LI-COR Odyssey imaging system was used to capture the image

f

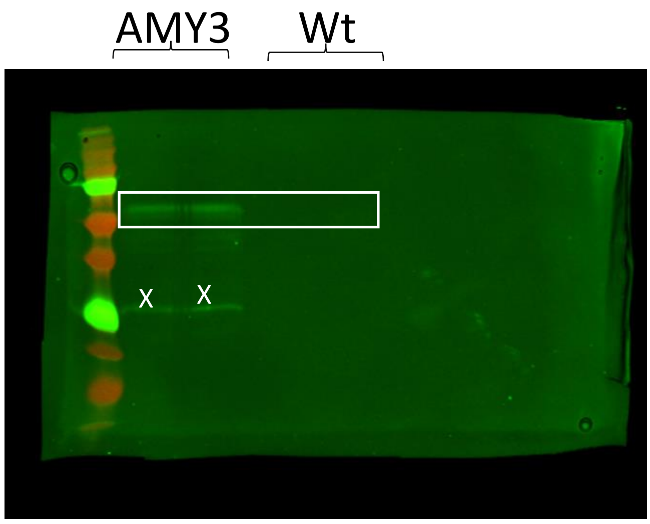

Raw image for western blot in Fig 3E, the EVs used in this data are from HEK AMY3 and HEK Wt cells. The antibody used in f is TSG101. LI-COR Odyssey imaging system was used to capture the image

g

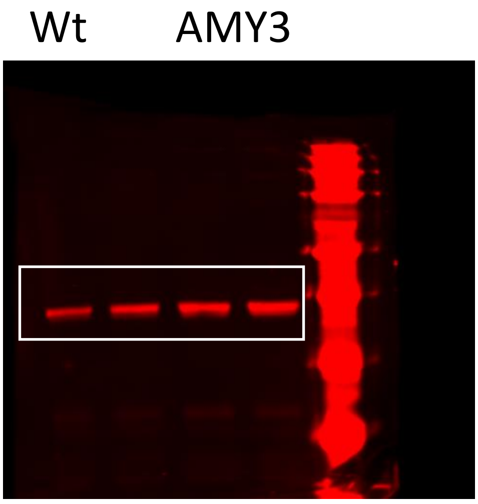

h

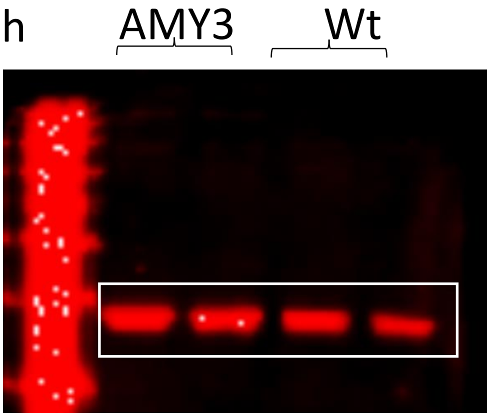

Raw images for western blot in Fig 5A, the EVs used in this data are from HETCTR and Wt mice. The antibody used in g, and h are TSG101 and HSP70, respectively. LI-COR Odyssey imaging system was used to capture the image

i

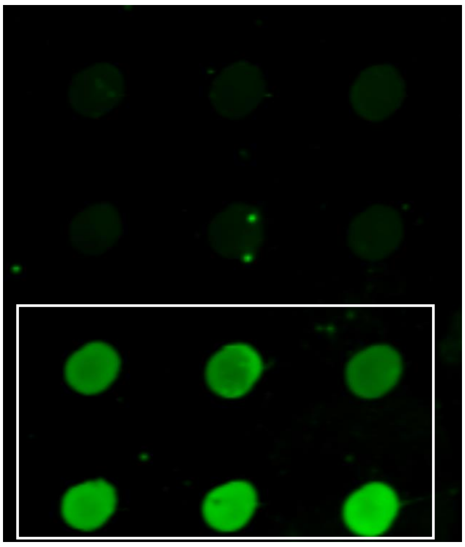

j

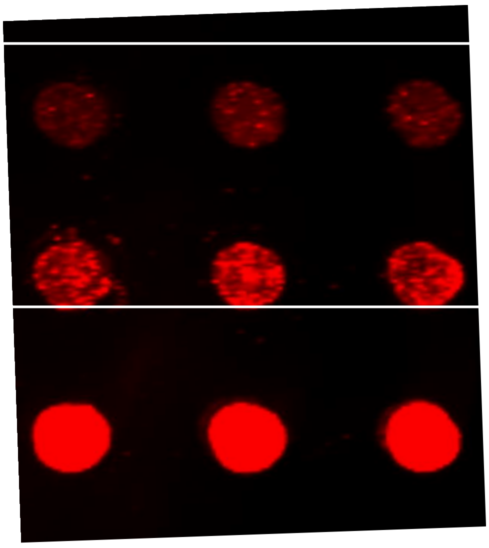

Raw images for dot blot in Fig 5A, the EVs used in this data are from mouse HET-CTR and wild type brains. The antibody used in i (green), and j (red) are CTR and 6E10 (amyloid beta), respectively. LI-COR Odyssey imaging system was used to capture the image

j

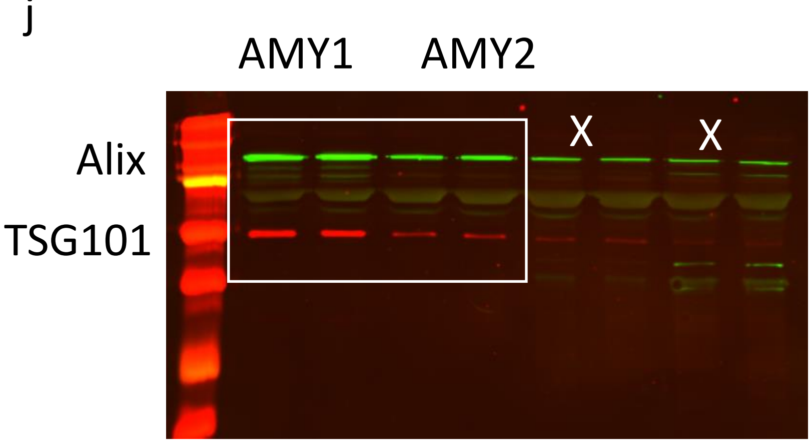

Raw images for western blot in Fig S1, the EVs used in this data are from HEK AMY1, and HEK AMY2 cells . The antibody used in j is Alix and TSG101, respectively. LI-COR Odyssey imaging system was used to capture the image
